# Inhibition of Inflammation by an Air-Based No-Ozone Cold Plasma in TNF-α-Induced Human Keratinocytes: An In Vitro Study

**DOI:** 10.3390/cimb48010084

**Published:** 2026-01-15

**Authors:** Byul-Bora Choi, Seung-Ah Park, Jeong-Hae Choi, Min-Kyeong Kim, Yoon Deok Choi, Hae Woong Lee, Gyoo-Cheon Kim

**Affiliations:** 1 Corporate Affiliated Research Institute, Feagle Co., Ltd., Yangsan 50561, Republic of Korea; cbbrstar@feagle.co.kr (B.-B.C.); pns610@feagle.co.kr (S.-A.P.); monday27@feagle.co.kr (J.-H.C.); 2 Department of Dentistry, College of Medicine, Kosin University, Busan 49267, Republic of Korea; black-71@hanmail.net; 3 Miul Dermatologic Clinic, 26, Centum dong-ro, Haeundae-gu, Busan 48059, Republic of Korea; miulskin@naver.com; 4 Louis Dermatologic Clinic, 72, Jangja-daero, Guri-si 11948, Republic of Korea; louiskin@naver.com; 5 Department of Oral Anatomy, School of Dentistry, Pusan National University, Yangsan 50612, Republic of Korea

**Keywords:** dermatitis, inflammation, keratinocyte, no-ozone cold plasma, skin

## Abstract

**Background/Objectives**: Recent studies have reported the effectiveness of cold plasma technology in treating skin inflammation and wounds. We investigated the effect of an air-based no-ozone cold plasma device (Air NCP) on the inflammatory response in human keratinocytes (HaCaT). **Methods**: The cytotoxicity of Air NCP was assessed using the sulforhodamine B assay, and its ozone concentration and operating temperature were measured to evaluate safety. To determine its anti-inflammatory effect, inflammation was induced with tumor necrosis factor-alpha (TNF-α), and changes in inflammation-related gene expression were analyzed using reverse transcription-polymerase chain reaction and Western blot analysis. The level of prostaglandin E2 (PGE2), an indicator of inflammation, was measured using an enzyme-linked immunosorbent assay. **Results**: Air NCP showed no cytotoxicity in HaCaT cells. Moreover, the expression of TNF-α, interleukin-6, and interleukin-1β significantly decreased following treatment (*p* < 0.001). The levels of phosphorylated nuclear factor kappa B and phosphorylated signal transducer and activator of transcription-3 were also reduced (*p* < 0.001). Western blot analysis further confirmed that inflammation-activated mitogen-activated protein kinase factors were reduced by Air NCP, while cyclooxygenase-2 and PGE2 levels similarly decreased. **Conclusions**: These results indicate that Air NCP treatment suppresses the expression of inflammatory mediators in skin inflammation, demonstrating a clear anti-inflammatory effect.

## 1. Introduction

Skin tissue acts as a barrier that protects the body from harmful external environments such as pathogens, ultraviolet rays, and air pollutants [1]. Skin keratinocytes, which account for >90% of the epidermis, come into contact with numerous antigens and are exposed to stressful conditions. They produce a range of inflammatory mediators, including cytokines and chemokines, that participate in inflammatory and immune responses [2,3]. Damage to the epithelial cells forming this barrier disrupts the protective function of the skin and leads to excessive secretion of inflammatory mediators, resulting in skin inflammation [4].

Treatment options such as anti-inflammatory drugs and antibiotics that relieve itching have been used to alleviate skin inflammation [5,6]. However, these drugs often cause side effects in patients with inflammatory skin diseases [7], with symptoms ranging from mild to systemic [8]. Dupilumab, a drug that relieves skin inflammation, is the only biological treatment approved by the U.S. Food and Drug Administration for severe atopic dermatitis in children and adults, yet studies on its safety remain limited. Dupilumab has been reported to cause ocular side effects such as conjunctivitis, dry eyes, itchy eyes, and keratitis; notably, studies involving patients with atopic dermatitis have shown that conjunctivitis is particularly common [9]. In addition, drugs containing ingredients such as adapalene, benzoyl peroxide, and tazarotene, which are used to treat inflammatory skin diseases, can also cause side effects, such as skin peeling, erythema, burning, stinging, and dryness [10]. In particular, in atopic dermatitis—a common inflammatory skin disease—it is difficult to use steroid-based drugs continuously in young children. Therefore, alternative treatment methods with fewer side effects or those that can prevent recurrence are needed [11,12,13].

One potential alternative treatment is plasma, which is the fourth state of matter, in which positive and negative charges are equally distributed. Plasma is created by ionizing a gas through heat or a strong electromagnetic field. The plasma formation process generates electrons, ions, active species, visible light, and electromagnetic fields. Among the various components generated by plasma, reactive oxygen species (ROS) and reactive nitrogen species (RNS) are the main active substances [14]. Its efficacy in treating skin diseases has recently been actively studied, and it is emerging as an innovative therapeutic technology in dermatology [15]. In a mouse model with induced atopic dermatitis, the severity of dermatitis and serum Ig E levels were reduced after 0.5 L/min argon plasma treatment [16]. Infiltration of mast cells and macrophages was reduced by an air discharge plasma device in these mice with induced atopic dermatitis [17].

Plasma is involved in wound healing and the repair of damaged skin tissue, resulting in improved collagen synthesis, angiogenesis, and epithelialization of the skin [18,19,20]. Moon et al. investigated the effects of plasma on atopic dermatitis and reported that plasma treatment for 2 weeks in a mouse model of atopic dermatitis induced by *Dermatophagoides farinae* extracts reduced the severity of dermatitis, transepidermal water loss, and serum immunoglobulin E levels compared with the untreated control group [16]. In addition, Lee et al. reported that plasma treatment in a mouse model of psoriasis induced by imiquimod suppressed the increase in the expression of pro-inflammatory molecules compared with that in untreated mice. Furthermore, the differentiation of Th17 cells, which play an important role in the development of psoriasis, was suppressed by plasma treatment, suggesting that plasma may be useful for treating CD4+ T cell-mediated autoimmune diseases, such as psoriasis [21]. While previous mouse model studies provide insight into the potential effects of plasma, their direct application to humans is limited. Therefore, more relevant data using human skin models or human keratinocytes are needed for translation to human applications. Plasma also has the potential to be applied in the treatment of patients with inflammatory skin diseases by strengthening the skin barrier function and altering the lipid composition of the skin [22].

However, despite its excellent efficacy, the plasma used in previous studies has been limited in development because of difficulties in reducing device size due to gas supply requirements. To generate plasma based on a specific gas, a gas supply is required, necessitating regular management, including gas replacement and storage. This poses a significant burden on individual safety management. To overcome these limitations, we developed an air-based no-ozone cold plasma device (Air NCP) with a wireless air source for ease of use. In this study, we aimed to verify whether this device was involved in alleviating and reducing skin inflammation by observing its effect on the inflammatory response in human keratinocytes (HaCaT).

## 2. Materials and Methods

### 2.1. Air NCP Device

**Figure 1:**
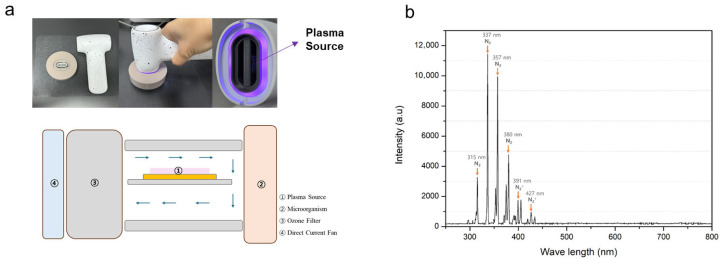
Characteristics of the air-based no-ozone cold plasma device (Air NCP) used in this experiment. (**a**) Image of the Air NCP. (**b**) Optical Emission Spectroscopy intensity emitted from the Air NCP.

The Air NCP (model code: AC-01) used in this study was developed by Feagle Co., Ltd. (Yangsan, Republic of Korea) and consists of a floating-electrode dielectric barrier discharge system (Figure 1a). Unlike conventional air plasma devices, this NCP system features a specialized design that enables the plasma generated from the air source to be effectively delivered to the cells while efficiently removing ozone gas. Figure 1a illustrates the airflow pathway of the NCP device. The dielectric material used was alumina, with dimensions of 20 mm (width) × 40 mm (height) × 0.7 mm (thickness). Both surfaces of the alumina were coated with silver paste electrodes applied via silk screen printing. The top electrode measured 5.5 mm^2^, and the bottom electrode was rectangular, measuring 8.4 mm (width) × 4 mm (height).

The main body of the device is equipped with a power button and can be operated for 1, 3, or 5 min. Plasma is generated and emitted from the front of the device, while the back houses a fan that circulates air internally. Using air as the working gas, the device was designed so that when the power button is pressed, air is drawn in and plasma is generated at an output voltage of 6.5 kVpp with a frequency of 20 kHz.

### 2.2. Optical Emission Spectroscopic Analysis of the Air NCP Device

Optical emission spectroscopy was performed to identify the active species generated by the Air NCP. The emission spectra were recorded over a wavelength range of 200–800 nm using an HR+ D1561 spectrometer (Ocean Optics, Dunedin, FL, USA), with an integration time of 1.0 s.

### 2.3. Temperature and Ozone Measurements of the Air NCP Device

The temperature at the plasma generation inlet was measured for 5 min using a contact temperature gun (CIT; Fluke Corporation, Everett, WA, USA) and verified using a handheld thermocouple during the final 5 min after plasma generation. The ozone concentration produced by the device was measured using a Serinus 10 ozone analyzer (OA; Acoem Ecotech, Knoxfield, VIC, Australia). To determine the real-time accumulated ozone level, both the fan side and the plasma generation component were operated for 1 min each. During measurement, the ozone meter nozzle was positioned as close as possible to the outlet, maintaining a distance of 2 mm.

### 2.4. Cell Culture

HaCaT cells were obtained from the Department of Oral Anatomy, School of Dentistry, Pusan National University and have been previously described [23]. HaCaT cells were cultured in Dulbecco’s Modified Eagle Medium (Gibco, Grand Island, NY, USA) supplemented with 10% fetal bovine serum (Gibco) and 1% penicillin–streptomycin (Gibco) and incubated at 37 °C in a humidified atmosphere containing 5% CO_2_. To establish an in vitro model of skin inflammation, HaCaT cells were treated with tumor necrosis factor-α (TNF-α; OriGene Technologies, Rockville, MD, USA) at a concentration of 0.1 µg/mL. After TNF-α treatment, the cells were exposed to the Air NCP for 1, 3, or 5 min and then incubated for 2 h.

### 2.5. Air NCP Treatment of Cells

A circular plastic support was fabricated to ensure stable plasma treatment for treating cells with Air NCP. The distance between the cells and the plasma source was approximately 20 mm, and cells were treated for 1, 3, and 5 min, respectively. The difference in effective effects for direct and indirect plasma treatment methods was confirmed by processing. Air NCP treatment was classified into direct and indirect methods as follows: Direct—TNF-α and Air NCP were applied to cells in a culture dish, followed by a 2 h incubation; Indirect—TNF-α and Air NCP were applied to cell-free medium in a separate dish, and the treated medium was subsequently transferred to a cell-containing dish.

### 2.6. Sulforhodamine B Assay

HaCaT cells were seeded in culture dishes at a density of 2 × 10^6^ cells/mL, incubated overnight at 37 °C, and then replaced with fresh medium. The cells were treated with the Air NCP for 1, 3, or 5 min and subsequently incubated for 24 h at 37 °C. The medium was removed, and the cells were washed three times with phosphate-buffered saline (Gibco). Next, 4% paraformaldehyde (Biosesang, Seoul, Republic of Korea) was added and incubated at room temperature for 30 min. The cells were then washed three times with distilled water, stained with 0.4% SRB solution (Sigma-Aldrich Corp., St. Louis, MO, USA), and incubated at room temperature for 30 min. After staining, the cells were washed five times with 1% acetic acid, and the bound SRB dye was dissolved in 10 mM Tris solution. The resulting supernatant was transferred to a 96-well plate, and absorbance was measured at 515 nm using a microplate reader (Multiskan GO, Thermo Scientific, Rockford, IL, USA).

### 2.7. Reverse Transcription-Polymerase Chain Reaction

HaCaT cells were seeded in 60 mm culture dishes at a density of 2 × 10^6^ cells/mL and cultured overnight in a 5% CO_2_ incubator. The medium was then replaced with one containing TNF-α at a concentration of 0.1 μg/mL, and the experimental groups were treated with the Air NCP for 1, 3, or 5 min, followed by incubation for 2 h. Both direct and indirect Air NCP methods were applied to the cells. Total RNA was extracted using TRIzol reagent (Invitrogen Life Technologies, Carlsbad, CA, USA). The concentration of the isolated RNA was quantified, and cDNA was synthesized using RT Master Mix (Invitrogen Life Technologies) with 2 µg of RNA. RT-PCR was conducted using the MG 2X HOT Mastermix with dye (MGmed, Seoul, Korea). PCR amplification was performed using a T100 Thermal Cycler (Bio-Rad, Hercules, CA, USA) with the following primers: TNF-α, 5′-CGAGTGACAAGCCTGTAGCC-3′ and 5′-CCCTTCTCCAGCTGGAAGAC-3′; interleukin (IL)-6, 5′-GATGGATGCTTCCAATCTGGA-3′ and 5′-AGCCACTGGTTCTGTGCCT-3′; IL-1β, 5′-ACAAAATACCTGTGGCCTTGG-3′ and 5′-GGTGCTGATGTACCAGTTGGG-3′; and GAPDH, 5′-ACTGGCATGGCCTTCCGT-3′ and 5′-CCACCCTGTTGCTGTAGCC-3′.

### 2.8. Western Blot Analysis

After washing the cells twice with phosphate-buffered saline, radioimmunoprecipitation assay buffer (Invitrogen Life Technologies) was added. The cells were incubated on ice for 10 min and then centrifuged at 12,000 rpm for 15 min at 4 °C, after which the supernatant was collected. The protein concentration of each sample was measured using the Bradford assay (Bio-Rad, Hercules, CA, USA). Proteins were separated by electrophoresis on a 10% sodium dodecyl sulfate–polyacrylamide gel and transferred to a polyvinylidene fluoride membrane (Millipore, Billerica, MA, USA). The membrane was blocked in Tris-buffered saline containing 5% skim milk for 1 h, and then incubated overnight at 4 °C with the following primary antibodies: TNF-α (1:1000), ERK (1:1000), phosphorylated (p)-ERK (1:1000), JNK (1:1000), p-JNK (1:1000), p-38 (1:1000), p-p38 (1:1000), GAPDH (1:2000) (Santa Cruz Biotechnology, Santa Cruz, CA), p-NF-κB (1:1000), NF-κB (1:1000), p-STAT3 (1:1000), STAT3 (1:1000), and COX-2 (1:1000) (Cell Signaling Technology, Danvers, MA, USA). After washing three times for 10 min each with Tris-buffered saline containing Tween 20 (TBS-T), the membranes were incubated with secondary antibodies for 2 h and then washed three times for 10 min each with TBS-T. Protein expression levels were visualized using an AI 680 imaging system (GE Healthcare, Uppsala, Sweden) after treatment with enhanced chemiluminescence reagents (Thermo Scientific).

### 2.9. Prostaglandin E2 Assay

The amount of PGE2 in the culture medium of the control and experimental groups was measured using a PGE2 enzyme-linked immunosorbent assay kit (R&D Systems, Minneapolis, MN, USA) according to the manufacturer’s protocol.

### 2.10. Statistical Analysis

Experimental results were analyzed using one-way or two-way analysis of variance followed by Tukey’s post hoc test with SPSS Statistics 20 software (IBM Corp., Armonk, NY, USA). All experiments were performed in triplicate (*n* = 3), and the results are presented as mean ± standard deviation.

## 3. Results

### 3.1. Air NCP OES

OES analysis was performed to determine the intensity and chemical composition of emissions from the Air NCP. The emission spectrum generated by the plasma source showed nitrogen (N_2_) emission bands at wavelengths of 315, 337, 357, 380, and 400 nm, and N_2_^+^ emission bands at 391 and 427 nm. Although ROS and RNS were not directly generated by the air-based plasma, peaks corresponding to reactive N_2_ and N_2_^+^, which are secondary N_2_ systems, were observed (Figure 1b).

### 3.2. Air NCP Temperature and Ozone Measurement

To confirm the safety of the Air NCP device for skin application, the plasma generation temperature and ozone levels produced were measured. The recorded temperatures were 21.3, 22.7, 21.4, 21.8, and 23.6 °C, with an average of 22.16 ± 0.98 °C (Table 1). The ozone concentration measured on the fan side was 0.008, 0.007, 0.006, 0.006, and 0.008 ppm, with an average of 0.007 ± 0.00045 ppm. At the plasma generation site, the measured ozone concentrations were 0.008, 0.006, 0.006, 0.006, and 0.006 ppm, with an average of 0.006 ± 0.0004 ppm (Table 2).

### 3.3. Effect of the Air NCP Device on HaCaT Cytotoxicity

**Figure 2:**
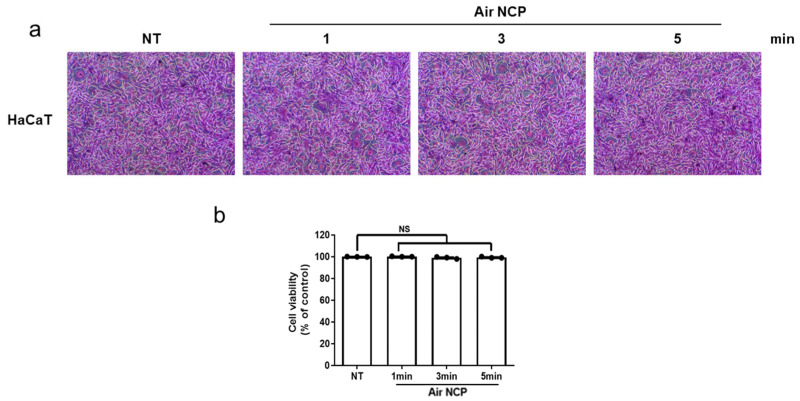
Cell viability analysis using sulforhodamine B (SRB) staining in human keratinocytes (HaCaT) treated with the Air NCP for 1, 3, and 5 min. (**a**) Microscopic images after SRB staining. Magnification: ×200 (**b**) Quantification of cell viability in Air NCP-treated cells using the SRB assay. NS: not significance. Black dot: Individual value plot.

Cell morphology observations (Figure 2) showed no visible damage in either the control or the Air NCP-treated groups. In addition, the absorbance values obtained after sulforhodamine B (SRB) staining were comparable between the experimental and control groups, indicating no significant cytotoxicity.

### 3.4. Effect of the Air NCP Device on the Expression of Inflammatory Cytokines

**Figure 3:**
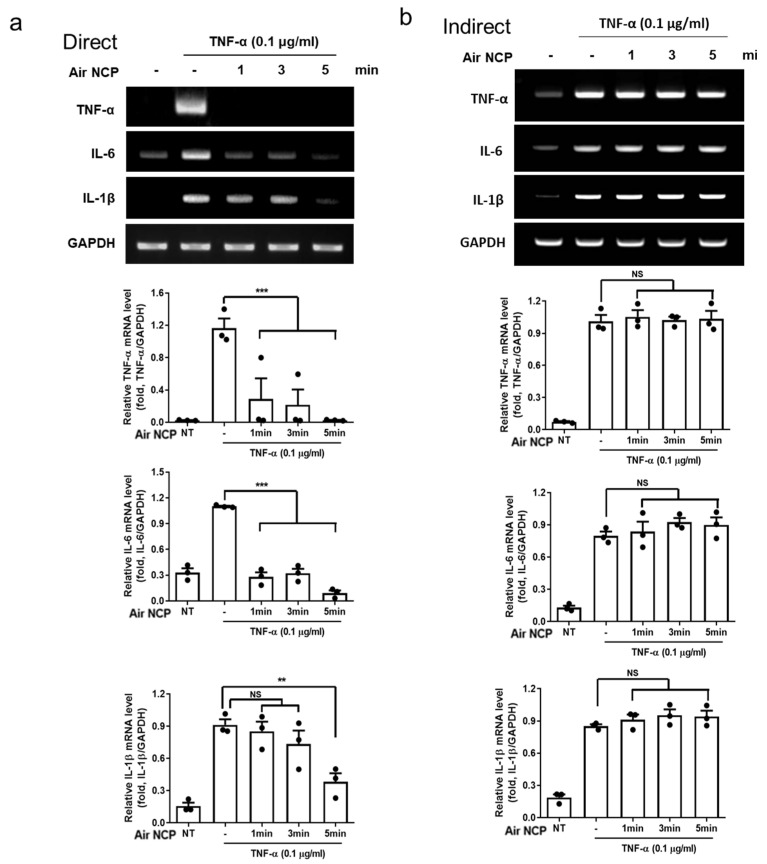
Anti-inflammatory effect of Air NCP in HaCaT cells induced by tumor necrosis factor-alpha (TNF-α), as analyzed by reverse transcription-polymerase chain reaction (RT-PCR). (**a**) After direct treatment with the Air NCP for 1, 3, and 5 min, the expression levels of TNF-α, Interleukin (IL)-6, and IL-1β were measured by RT-PCR. (**b**) After indirect treatment with the Air NCP for 1, 3, and 5 min, the expression levels of TNF-α, IL-6, and IL-1β were measured by RT-PCR. ** *p* < 0.01, *** *p* < 0.001.

Quantification analysis of expression levels using reverse transcription-polymerase chain reaction (RT-PCR) revealed that, after inflammation was induced in the cells, tumor necrosis factor-alpha (TNF-α) levels significantly decreased in the group directly treated with the Air NCP for 1 min. Interleukin (IL)-6 levels also gradually decreased with longer Air NCP treatment durations, showing a significant reduction in the group treated for 5 min (*p* < 0.001). Although the levels of IL-1β, another inflammatory cytokine, did not decrease after 1 min of treatment, its expression was reduced in the groups treated with the Air NCP for 3 and 5 min compared with the untreated group (Figure 3a). In contrast, inflammation was induced in the group treated indirectly with the Air NCP; however, no significant reduction pattern was observed after treatment (Figure 3b).

### 3.5. Effect of the Air NCP on Inflammation-Mediated Signaling

**Figure 4:**
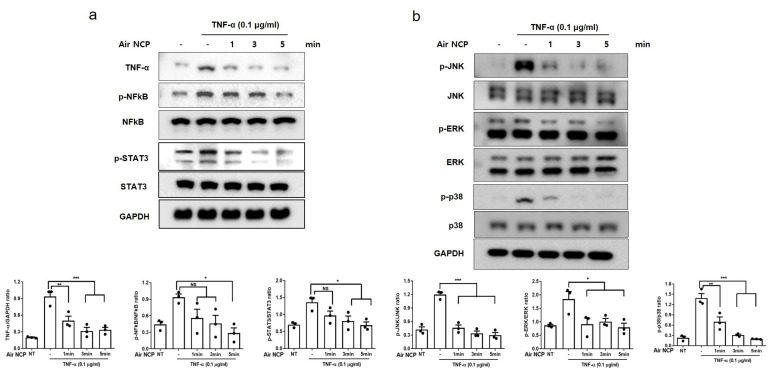
Anti-inflammatory effect of Air NCP in HaCaT cells induced by TNF-α, analyzed by Western blot. (**a**) Western blot analysis of TNF-α, phosphorylated (p)-nuclear factor kappa B, and p-signal transducer and activator of transcription 3 after treatment of HaCaT cells with Air NCP for 1, 3, and 5 min. (**b**) Western blot analysis of p-extracellular signal-regulated kinase, p-c-Jun N-terminal kinase, and p-p38 after treatment of HaCaT cells with Air NCP for 1, 3, and 5 min. * *p* < 0.05, ** *p* < 0.01, *** *p* < 0.001.

Western blot analysis revealed that TNF-α levels, which increased following TNF-α treatment, were slightly reduced by Air NCP treatment. The phosphorylation of the inflammatory transcription factors nuclear factor kappa B (NF-κB) and signal transducer and activator of transcription (STAT) also decreased in a treatment time-dependent manner (*p* < 0.001) (Figure 4a). These results were further supported by quantified data showing significant reductions in protein expression levels. In addition, the phosphorylation of mitogen-activated protein kinase (MAPK) pathway components—extracellular signal-regulated kinase (ERK), c-Jun N-terminal kinase (JNK), and p38—was significantly increased during inflammation but showed a decreasing trend after treatment with the Air NCP (Figure 4b).

### 3.6. Effects of Air NCP on Cyclooxygenase-2 and Prostaglandin E2 Production

**Figure 5:**
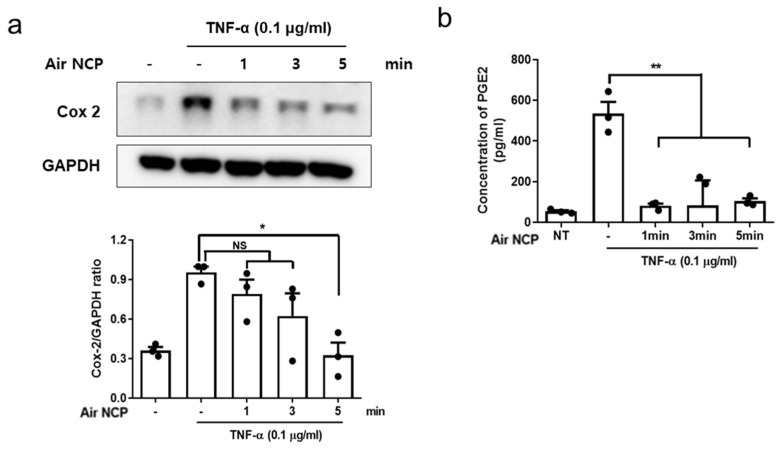
Air NCP inhibits cycloxygenase-2 (COX-2) and Prostaglandin E2 (PGE2) production in HaCaT cells through an anti-inflammatory effect. (**a**) Western blot analysis of COX-2 after treatment of HaCaT cells with Air NCP for 1, 3, and 5 min. (**b**) Concentrations of PGE2 were measured after treatment of HaCaT cells with Air NCP for 1, 3, and 5 min. * *p* < 0.05, ** *p* < 0.01.

Western blot assay confirmed that cyclooxygenase-2 (COX-2) expression increased in cells with induced inflammation and subsequently decreased in a time-dependent manner after treatment with Air NCP (Figure 5a). Prostaglandin E2 (PGE2) levels were higher in the cell supernatants following TNF-α exposure compared with those in the control group, and were significantly reduced in all groups treated with Air NCP (Figure 5b).

## 4. Discussion

Keratinocytes, the epithelial cells of the skin, respond to external environmental stimuli such as ultraviolet radiation or chemical agents by releasing inflammatory cytokines, including IL-1 and TNF-α, as well as factors such as IL-10 and IL-12 that regulate cellular immunity [24]. Keratinocytes, Langerhans cells, and melanocytes present in the epidermis produce slightly different cytokines during inflammation. Among these, IL-1β, IL-6, and TNF-α are mainly expressed by epidermal cells. In particular, TNF-α, a major pro-inflammatory cytokine, is known as a multifunctional mediator involved in immune regulation, inflammation, and cell proliferation [25,26]. TNF-α also induces extensive chemokine production to attract neutrophils, macrophages, and skin-specific memory T-cells, thereby modulating innate immune and inflammatory responses. These responses mediated by TNF-α are involved in the pathogenesis of contact dermatitis, including atopy, psoriasis, and allergies [27]. In this study, we investigated whether the Air NCP, a compact device utilizing plasma technology, could inhibit these responses in a cell inflammation model. Previous studies have demonstrated that plasma can reduce the expression of inflammatory factors elevated in skin inflammation models and promote the regeneration and repair of damaged skin tissue [28,29]. In this study, we found that the use of the Air NCP did not affect cell morphology or viability. These results confirm that the Air NCP, which generates cold plasma, does not cause thermal damage to cells, indicating its potential for safe use in humans.

The effects of plasma treatment on cells vary depending on whether the exposure is direct or indirect. Direct plasma treatment can influence cellular structures through interactions with charged particles such as electrons and ions, whereas indirect plasma treatment predominantly acts through reactive species, particularly ROS, generated by the plasma.

We also analyzed the mRNA expression levels of inflammatory cytokines to evaluate the anti-inflammatory effect of Air NCP treatment in the inflammation model. In the groups treated with the Air NCP, the expression of TNF-α, IL-6, and IL-1β decreased in a treatment-dependent manner, indicating that the Air NCP can mitigate the inflammatory response. Direct plasma treatment reduced pro-inflammatory cytokines such as TNF-α, IL-6, and IL-1β in inflammation-induced cells, whereas indirect treatment did not produce a similar effect. The anti-inflammatory response observed in direct plasma treatment may result from the effects of electrons or charged particles rather than the reactive substances themselves, meriting further investigation.

Furthermore, the protein levels of TNF-α, p-NF-κB, and phosphorylated STAT3 (p-STAT3) decreased in a time-dependent manner following Air NCP treatment. Since p-NF-κB and STAT3 are known to promote inflammatory skin response along with TNF-α, these findings indicate that the Air NCP alleviates inflammation by regulating inflammatory transcription factors. These results are consistent with a previous in vitro study showing that N_2_ plasma treatment of keratinocytes inhibited TNF-α/interferon-γ-induced activation of NF-κB and STAT1 and STAT3 [30]. Unfortunately, NF-κB activity has been primarily assessed using its phosphorylation status, so further validation using IκBα degradation or p65 nuclear translocation is required to demonstrate pathway inhibition comprehensively.

The MAPK pathway is a signaling cascade that transmits external stimuli to the cell nucleus, regulating cell proliferation, differentiation, and inflammatory responses [31]. The key MAPKs—ERK1/2, p38, and JNK—control the transcription and translation of inflammatory cytokines [32]. Additionally, they play an important role in activating NF-κB by promoting its translocation from the cytoplasm to the nucleus and enhancing its activity [33]. Air NCP inhibited inflammation-induced cellular responses and reduced the expression of ERK, p38, and JNK associated with MAPK.

To further verify the alleviating effect of Air NCP treatment on inflammation-induced cells, we analyzed changes in PGE2 concentration, an inflammatory mediator. Prostaglandins are eicosanoids involved in inflammation, platelet aggregation, and neurotransmitter secretion and are produced by various cell types [34]. PGE2 is the best-known mediator of inflammatory response and a lipid compound that is not stored within cells but induces the production of matrix metalloproteinases in inflammatory diseases that cause tissue damage [35]. Moreover, COX-2 is a key enzyme in the prostaglandin synthesis pathway that catalyzes the conversion of arachidonic acid into PGE2 [36]. In the group treated with Air NCP, COX-2 expression was lower than that in the inflammation-induced group. The HaCaT cells were exposed to media containing TNF-α, and PGE2 levels in the culture medium increased markedly compared with the control group. In contrast, PGE2 levels were significantly reduced in all groups treated with Air NCP.

These results show that the Air NCP treatment promotes the recovery of skin cells damaged by inflammation, suggesting its potential for treating inflammation-induced skin injury in the future. Although further studies are required to validate the therapeutic potential of Air NCP, the present findings provide foundational evidence of its anti-inflammatory effect on skin cells. Although the anti-inflammatory effect of Air NCP on human keratinocytes was confirmed, plasma active species could not be quantified. Additionally, the safety evaluation of the cells was based on short-term measurements, so further analyses are required. An evaluation considering the potential clinical applicability is needed.

## 5. Conclusions

In summary, a significant reduction was observed in the expression of inflammatory cytokines and PGE2 in the HaCaT inflammation model following treatment with Air NCP. While these findings suggest that this anti-inflammatory effect stems from the direct action of Air NCP, further research is needed. Therefore, future preclinical and clinical validation studies should develop Air NCP as a device that alleviates inflammation in inflammatory skin diseases and promotes the recovery and proliferation of tissues damaged by inflammation.

## 6. Patents

Four patents have been applied for and registered by Feagle Co., Ltd., including Korean registration (10-2572882), international application (PCT/KR2021/020248), U.S. application (18/270,838), and a second Korean application (10-2024-0113263).

## Figures and Tables

**Table 1:** Temperature of Air NCP device used in the experiments.

	1	2	3	4	5	Average
Temperature (°C)	21.3	22.7	21.4	21.8	23.6	22.16 ± 0.98 °C

**Table 2:** Ozone level of Air NCP device used in the experiments.

	1	2	3	4	5	Average
O_3_ level (ppm ^a^)	0.008	0.006	0.006	0.006	0.006	0.006 ± 0.004 ppm

^a^ parts per million (ppm).

## Data Availability

The original contributions presented in this study are included in the article. Further inquiries can be directed to the corresponding authors.

## Abbreviations

The following abbreviations are used in this manuscript:

HaCaT	Human keratinocytes
NF-κB	Nuclear factor kappa B
Air NCP	Non-ozone cold plasma device based on a wireless air source
p-NF-κB	Phosphorylated NF-κB
p-STAT3	Phosphorylated STAT3
PGE2	Prostaglandin E2
RNS	Reactive nitrogen species
ROS	Reactive oxygen species
RT-PCR	Reverse transcription polymerase chain reaction
STAT3	Signal transducer and activator of transcription 3
SRB	Sulforhodamine B
TBS-T	Tris-buffered saline with Tween 20
TNF	Tumor necrosis factor
